# Analysis of the Chloroplast Genome of *Ficus simplicissima* Lour Collected in Vietnam and Proposed Barcodes for Identifying *Ficus* Plants

**DOI:** 10.3390/cimb45020067

**Published:** 2023-01-27

**Authors:** Thuy Thi Thu Vu, Lien Thi Kim Vu, Lam Tung Le, Thu Thi Mai Lo, Mau Hoang Chu

**Affiliations:** 1 Department of Genetics and Biotechnology, TNU-University of Education, Thainguyen 250000, Vietnam; 2 Institute of Theoretical and Applied Research, Duy Tan University, Hanoi 100000, Vietnam; 3 Faculty of Natural Sciences, Duy Tan University, Da Nang 550000, Vietnam; 4 VAST Institute of Biotechnology, Hanoi 100000, Vietnam; 5 Department of Biology, Taybac University, Sonla 360000, Vietnam

**Keywords:** chloroplast genome, DNA barcode, *Ficus simplicissima*, medicinal plants, phylogeny

## Abstract

*Ficus simplicissima* Lour. is an Asian species of fig tree in the family Moraceae. The chloroplast (cp) genome of *F. simplicissima* m3 was sequenced using the Pacbio sequel platform. The *F. simplicissima* cpDNA has a size of 160,321 bp in length, of which GC content accounts for 36.13%. The cp genome of *F. simplicissima* consists of a single large copy (LSC) with a size of 91,346 bp, a single small copy (SSC) with a size of 20,131 bp, and a pair of inverted repeats with a size of 24,421 to 24,423 bp. The cp genome of *F. simplicissima* has 127 genes, including 85 protein-coding genes, eight rRNA genes, and 34 tRNA genes; 92 simple sequence repeats and 39 long repeats were detected in the cpDNA of *F. simplicissim*. A comparative cp genome analysis among six species in the *Ficus* genus indicated that the genome structure and gene content were highly conserved. The non-coding regions show more differentiation than the coding regions, and the LSC and SSC regions show more differences than the inverted repeat regions. Phylogenetic analysis supported that *F. simplicissima* m3 had a close relationship with *F. hirta*. The complete cp genome of *F. simplicissima* was proposed as a chloroplast DNA barcoding for genus-level in the Moraceae family and the *psbA-trnH* gene region for species-level identification.

## 1. Introduction

The chloroplast (cp) genome is circular and relatively conserved among plants in terms of size, structure, and gene content [1]. The cp genome generally comprises two copies of inverted regions that divide the genome into a large single-copy region (LSC) and a small single-copy region (SSC). The cp genome provides genes participating in photosynthesis, transcription, and translation. In addition, its non-coding intergenic spacer regions are highly conserved and can be used for phylogenetics, population genetics, and species identification [2]. Earlier research used partial cp sequences for plant barcoding [3], but they are only universal for some plant taxa and have limitations at lower taxonomic levels [4,5]. Therefore, the whole cp genome is informative and extensive for plant barcoding [6,7]. With the advance of next-generation sequencing, whole chloroplast genome acquisition is now simpler and faster than ever. Coverage of four cp junctions between the inverted repeat (IR) and single-copy regions is performed by a third–generation sequencer, the PacBio system with Single Molecule Real-Time (SMRT) technology [8].

*Ficus simplicissima* Lour. is a species of fig tree in the *Ficus* genus. This genus contains about 1000 species of trees, shrubs, and vines and is distinct with a unique fruit structure known as syconium [9]. The species is found predominantly native to East Asian tropical regions. In folk medicine, *F. simplicissima* was used to treat pneumonia, vitiligo, diarrhea, tonsillitis, cough, and rheumatic pain and promote lactation [10]. Moreover, combining modified Radix Fici Simplicissimae with Western medicines was considered a potential treatment for SARS-CoV-2 patients [11].

Although the *Ficus* genus has different applications, its phylogenetic relationship is controversial. The *Ficus* classification system was based on morphological characteristics, and analysis of ribosomal DNA was reported by Corner (1965) and Huang et al. (2022). The information on the cp genomes of *Ficus* can be used in species identification and phylogenetic analysis of *Ficus* species [12,13]. However, the cp genome of *F. simplicissima* Lour. needed more because there were only raw reads of the cp genome of *F. simplicissima* Lour. at the China National GenBank [13].

The purpose of this study was to sequence and annotate the cp genome of *F. simplicissima* Lour m3; cp genome analysis, evaluate diversity and molecular evolutionary analysis of *Ficus* genus in the Moraceae family, search for cpDNA markers as potential DNA barcodes for *Ficus* species identification.

## 2. Materials and Methods

### 2.1. Plant Material, DNA Extraction, and cp Genome Sequencing

**Figure 1:**
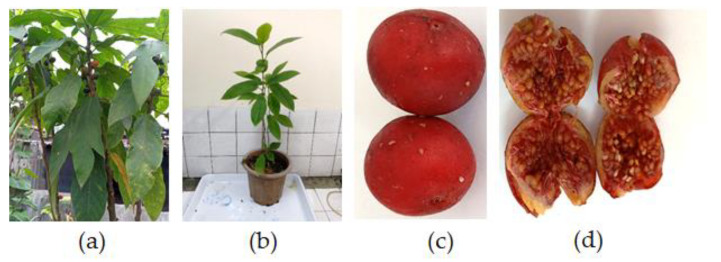
Sample image of *Ficus simplicissima* Lour. m3 regenerated from seeds collected in the Thai Nguyen province (Hung Son township, Dai Tu district). (**a**) Plants regenerated in vitro from seeds grown in the experimental garden and (**b**) grown in pots, they are kept at the Department of Biology, Thai Nguyen University of Education; (**c**,**d**) fruit of *F. simplicissima*.

*Ficus simplicissima* Lour. m3 seeds were collected in the Thai Nguyen province (Hung Son township, Dai Tu district), then cultured on Murashige and Skoog medium. Regeneration of plants in vitro and plants grown in pots and in experimental gardens. *Ficus simplicissima* samples were identified by comparative morphology by the Department of Botany, Thai Nguyen University of Education, Vietnam (Figure 1). Fresh leaves from plantlets were harvested for genomic DNA extraction using the DNeasy^®^ Plant Mini Kit. Absorption spectroscopy analysis on a Shimadzu Biospec Nano instrument at wavelengths A260 and A230 assessed DNA sample purity.

The concentration of DNA was determined using a Qubit 3 Fluorometer and Qubit HS DNA reagents. The integrity of the gDNA is assessed by 0.8% agarose gel electrophoresis. Besides, the total genomic DNA was used for library preparation using SMRTbell Express Template Prep Kit 2.0 (Pacific Biosciences, Menlo Park, CA, USA) following the manufacturer’s protocol (Pacific Biosciences). SMRTbell libraries were loaded on one chip and sequenced on a Pacbio SEQUEL system at the Key Laboratory for Gene Technology, Institute of Biotechnology (Hanoi, Vietnam).

### 2.2. Genome Assembly and Annotation

The cp genome sequences were determined via pbmm2 (https://github.com/PacificBiosciences/pbmm2, accessed on 20 August 2022) by mapping to *Ficus hirta* cp genomes (NC_051532.1) as the reference. Afterward, the CANU program [14] was used to assemble the cp genome. The assembled genome was annotated and analyzed using the GeSeq tool [15]. The tRNAscan-SE ve has confirmed the tRNA genes with default parameters. 1.21 software [16]. A circular genome map was created by the OrganellarGenomeDRAW tool (OGDRAW) ver. 1.3.1 [17]. Two methods searched Repeat sequences. MISA-web [18] was used to detect microsatellites with the following setting: 10 repeat units for mono-, five repeat units for di, four repeat units for tri-, and three repeat units for tetra-, penta-, and hexanucleotide SSRs. Dispersed repeats (including forward, reverse, complement, and palindromic repeats) were identified by REPuter [19] with a minimum repetition size of 20 times, hamming distance = 3, and sequence identities ≥90%.

### 2.3. Genome Comparison

For comparative analysis, five *Ficus* cp genomes were obtained from NCBI, and their accession numbers are as follows: *Ficus concinna* (MZ128521), *Ficus formosana* (NC_059898), *Ficus pandurata* var. angustifolia (NC_063593), *Ficus sarmentosa* voucher ZZ063 (NC_061976), *Ficus hirta* (NC_051532). The cp DNA nucleotide sequences of the six *Ficus* species, including the genome of *F. simplicissima* m3, have been linked with the MAFFT server and visualized by mVISTA software (Shuffle-LAGAN mode) [20] and used the *F. simplicissima* m3 genome to compare with genome five remaining species. Large single-copy (LSC), small single-copy (SSC), and inverted repeat (IR) regions among the *Ficus* species were visualized as the junction sites of chloroplast genomes and compared using the IRscope online program. Codon usage trends, Pi values, and nucleotide sequence polymorphisms among six *Ficus* species were determined by calculating the pi sliding window analysis between cp DNAs in DnaSP ver. 6.12.03 [21]. We chose a window size of 600 bp with a step size of 200 bp for sequence divergence analysis.

### 2.4. Phylogenetic Identification

The sequences of *psbA-trnH* and complete cp genome were downloaded from the GenBank of NCBI to illustrate phylogenetic relationship and position. The MAFFT server [22] was applied to align these sequences and maximum likelihood trees with 1000 bootstrap replicates were constructed by FastTree version 2.1.11 [23]. Subsequently, FigTree (version 1.4.4) [24] was employed to visualize phylogenetic trees.

## 3. Results

### 3.1. Chloroplast Genome Features of Ficus Simplicissima m3

**Figure 2:**
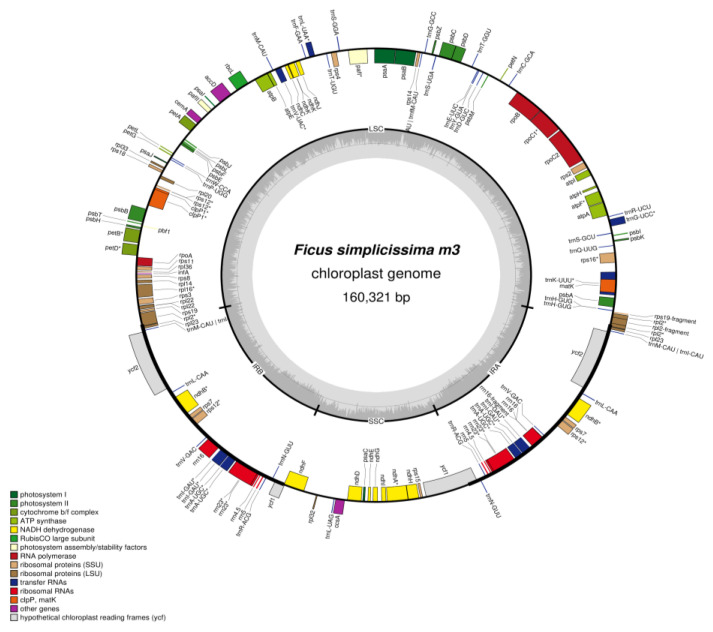
Circular map of the chloroplast genome of *F. simplicissima* m3. Genes located outside of the circle are transcribed counter-clockwise, while genes shown inside are transcribed clockwise. The darker gray in the inner circle indicates GC content, and the lighter gray corresponds to AT content. Genes marked with the sign ‘*’ are the gene with intron.

In total, 51,578 reads and 3.7 Gb raw data sequences of the whole genome were generated from *F. simplicissima* m3. After trimming and selecting reads, the *F. simplicissima* m3 cp genome with a size 160,321 bp was assembled. The *Ficus* plastome possessed the classic quadripartite structure (Figure 2), containing one LSC region (68,977 bp), one SSC region (20,131 bp) and two inverted repeat (IR) regions (24,421 and 24,423 bp). The overall GC content was 35.9 (%) (Table 1). The cp genome of *F. simplicissima* m3 contains 127 genes, including 85 protein-coding genes, 8 rRNA genes, 34 tRNA genes. Of these, 18 genes were duplicated in the IR region and 21 genes contain introns. Additionally, 11 protein-coding genes (*rps16*, *petB*, *petD*, *atpF*, *ndhA*, *ndhB* (× 2), *rpoC1*, *rpl16 and rpl2* (× 2)) and six RNA genes (*rrn23* (×2), *trnI-GAU* (× 2) and *trnA-UGC* (× 2)) contained only one intron, while two protein-coding genes (*clpP and ycf3*) had two introns (Supplementary File S1 Table S1). The complete chloroplast genome of the *F. simplicissima* was submitted to GenBank in November 2022 and was granted the code BankIt2647431 *Ficus_simplicissima*_m3 OP928145 on 5 December 2022, and is waiting for the accession number (Supplementary File S2).

### 3.2. Codon Usage

The chloroplast genome of *F. simplicissima* was analyzed for its codon usage frequency based on the nucleotide sequence of protein-coding genes and on relative synonymous codon usage (RSCU). The relative frequency of synonymous codons of the *F. simplicissima* m3 cp coding sequence was estimated. The results indicate that protein-coding genes were encoded by 54,960 codons and the four most common codons were UUU (phenylalanine), AAA (lysine), AAU (asparagine), and AUU (Isoleucine), corresponding to 2393 (4.35%), 2287 (4.16%), 1967 (3.57%), and 1830 (3.32%) codons, respectively. In terms of the prevalence of translated amino acids, leucine (5719) and isoleucine (4569) were the two most frequently used amino acids, while the least abundant was tryptophan (692 codons, approximately 1.25%). Thirty codons were used more frequently than other synonymous codons (RSCU > 1) and thirty-two codons were considered as relatively less used codons (RSCU < 1). Furthermore, AUG and UGG (methionine and tryptophan) showed no bias (RSCU = 1).

### 3.3. Repeat Sequence Analysis

**Figure 3:**
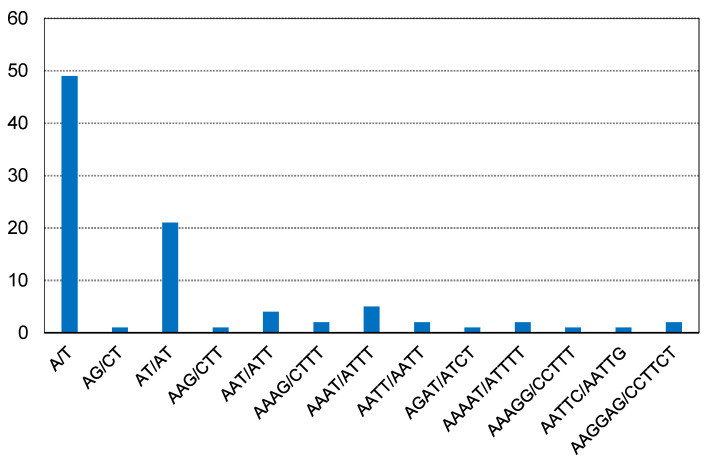
Number of different repeat units of SSRs.

Simple sequence repeats (SSRs) are tandemly repeated DNA sequences consisting of short, tandemly repeated di-, tri-, tetra-or penta-nucleotide motifs [25]. A total of 92 SSRs were identified in the *F. simplicissima* m3 cp genome. Among them, there were 49 mononucleotide repeats, 22 dinucleotide repeats, 5 trinucleotide repeats, 10 tetranucleotide repeats, 4 pentanucleotide, and 2 hexanucleotides. In addition, all the mononucleotide repeats belonged to A/T and were the highest (Figure 3).

Additionally, the long complex repetitive sequences were explored, containing forward repeats, reverse repeats, palindromic repeats and complement repeats in the *F. simplicissima* m3 chloroplast genome. We identified 11 forward, 1 reverse, 1 complement, and 26 palindromic repeats (Supplementary File S1 Figure S1). 

### 3.4. Phylogenetic Analysis

**Figure 4:**
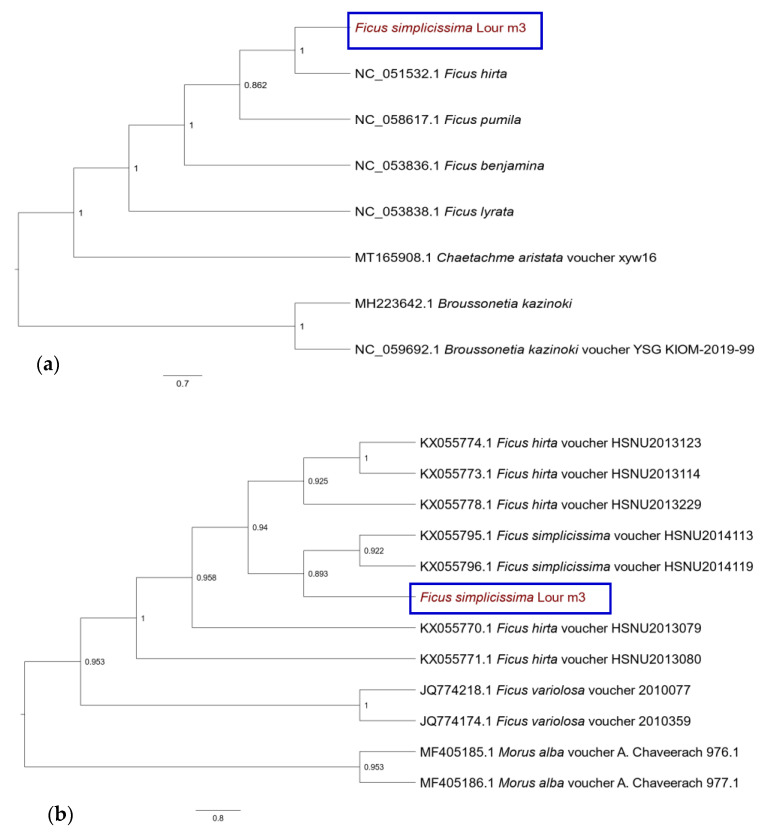
Phylogenetic relationships among Moraceae family based on complete cp genome (**a**) and *psbA-trnH* intergenic region (**b**).

To examine the phylogenetic relationships within the *Ficus* genus and Moraceae family, ML analysis was constructed based on the similarity of chloroplast sequences and the *psbA-trnH* intergenic region. As illustrated in the complete cp genome (Figure 4a), species are divided into groups on the phylogenetic tree with high bootstrap values (86.20–100%). The studied *F. simplicissima* m3 and *F. hirta* (Accession number in GenBank: NC_051532.1) were located in one group with 100% support. The branch support value (86.2%) for *F. pumila* (NC_058617.1) and this group was lower than the other branch support values. *F. benjamina* (NC_053836.1) and *F. lyrata* (NC_053838.1) formed a well-supported monophyletic group.

The phylogenetic tree inferred from the *psbA-trnH* data displayed six distinct groups with strong support, with the support values ranging from 89.3 to 100% (Figure 4b). Group I contained two species of outgroup *Morus alba* (voucher *A. chaveerach* 976.1, (Accession number in GenBank: MF405185.1 and voucher *A. chaveerach* 977.1, MF405186.1). The selected species belonging to the *Ficus* genus generated the five remaining groups. Almost all individuals in the same species fell into the same clade, except five *F. hirta* voucher. Group II included two vouchers of *F. variolosa* species (JQ774218.1 and JQ774174.1) with 100% bootstrap value. Three *F. simplicissima* species constituted group III, in which *F. simplicissima* voucher HSNU2014113 (KX055795.1) and *F. simplicisima* voucher HSNU2014119 (KX055795.1) formed one clade (bootstrap value = 92.2%) resolved as sister to the studied *F. simplicissima* m3 (bootstrap value = 89.3%). Five *F. hirta* species split into three separate groups. Group IV comprised two clades, one clade encompassed *F. hirta* voucher HSNU2013079 (KX055770.1) and *F. hirta* voucher HSNU2013080 (KX055773.1) with bootstrap value = 100, the rest were sister to *F. hirta* voucher HSNU2013229 (KX055778.1) with 92.5% branch support value. Three selected *F. simplicissima* species were the most closely related to three *F. hirta* species (KX0055774.1; KX055773.1; KX055778) in group V with a 94% bootstrap value.

### 3.5. Comparative Genomic Analysis

**Figure 5:**
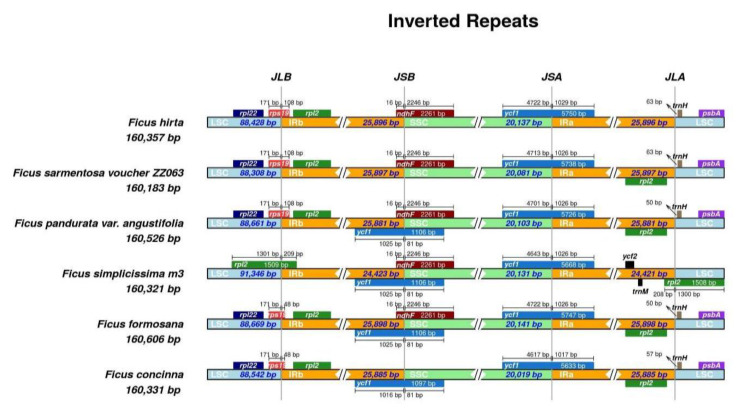
Contiguous positions between LSC, SSC, and IR regions of six *Ficus* cp genomes.

The junction sites in these cp genomes were relatively conserved. The length of IR ranged from 24,421 to 25,898 bp. Most of these species had a length of the LSC region of approximately 88,500 bp. By contrast, *F. simplicissima* m3 exhibited a larger LSC region of 91,346 bp (Figure 5) and the large single-copy (LSC) region of chloroplast DNA is highly efficient in species identification [26].

The rps19 gene is located in the junction region between LSC and IRb (JLB), while the *rpl2* gene covered this location in *F. simplicissima* m3. The *trnH* gene was shifted from JLA from 50 to 63 bp, except for *F. simplicissima* m3. The *ycf1* gene was found to have crossed the junction located in JSA and JSB, while it was absent in the JSB of *F. hirta* and *F. sarmentosa.* The *ndhF* gene covered the IRB-SSC region with a similarity size of 2261 bp. However, the *ndhF* gene was not observed in this region of *F. concinna* (Figure 5).

**Figure 6:**
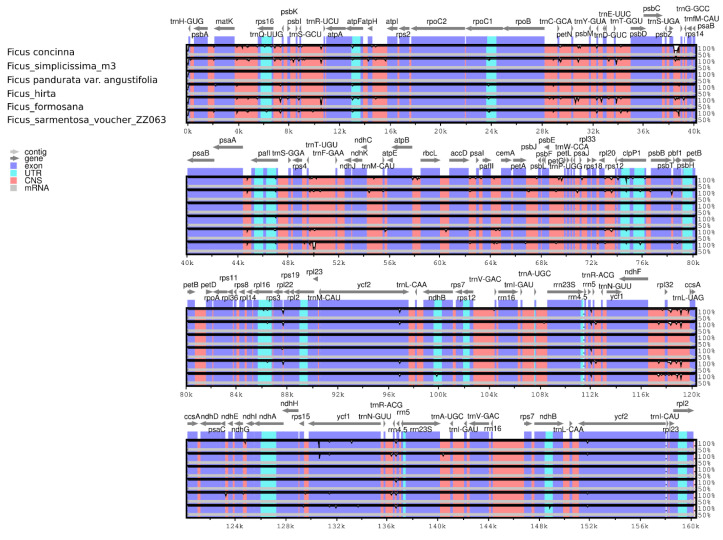
Sequence alignment of chloroplast genomes among six *Ficus* species. The *x*-axis shows the coordinates in the cp genome. The *y*-axis shows the recognition percentage (50% to 100%).

The *F. simplicissima* m3 cp genome was used as a reference to analyze the cp genome identity of the six *Ficus* species (Figure 6). The non-coding regions were supposed to be more divergent than the coding regions. A considerable number of variations were found including *ycf1*, *rpoC2*, *rpoC1*, *ycf2*, *ndhF*, *rps16—trnQ-UUG*, *trnS-UGA—trnG-GCC*, *trnT-UGU—trnF-GAA*, *petN—psbM*, *trnT-GGU—psbD*, *rpl32—trnL-UAG* in the intergenic spacer regions.

Nucleotide diversity in LSC and SSC regions was significantly higher than that in the IR regions (Figure 6). The Pi value among six *Ficus* species ranged from 0 to 0.01701, with an average of 0.00306. The results showed that six highly variable regions were detected consisting of *rps16—trnQ-UUG*, *trnC-GCA*, *rps14—psaB*, *clpB—psbB*, *trnL—ccsA—ndhD*, *rrn23S* (Figure 6). Among divergent regions, five belonged to the LSC and SSC regions, only one was located in the IRa region.

## 4. Discussion

The gene content and genomic organization of the *Ficus* cp genomes were highly conserved, and no rearrangement had been found. The *F. simplicissima* m3 cp genome was 160,321 bp in length and had a typical quadripartite structure including an SSC, and an LSC as well as a pair of IRs. The studied cp genome sequence showed a bias toward a higher A/T ratio in composition. The GC content of the IR regions was higher than that of the non-coding intergenic regions because of the presence of rRNA genes [27]. In addition, the number of predicted genes was smaller (127) than that (131) which were previously reported in the *F. simplicissima* [13], *F. concinna* [28], and *Broussonetia* species [29] genomes with 15 intron-containing genes. Several genes are known to possess structural intron variation, such as *atpF*, *rpoC2*, *rpl12*, *rps12*, and *rps16* [2,30]. The cp genome loses and gains introns during evolution, which plays a significant role in regulating gene expression via the alternative splicing or the stabilization of the transcript [31]. In the *F. simplicissima* m3 plastome, both *clpP* and *ycf3* genes contained double introns, while 11 other protein-coding genes, six rrRNA genes and five tRNA genes contain one intron.

In this study, the use of codons in the nucleotide sequence of protein-coding genes of the chloroplast genome of *F. simplicissima* tends to be specific codons used more frequently than other synonymous codons. This result was consistent with previous related reports [32,33,34,35,36]. The level of use of codons varies between individuals within species and between species of the genus. RSCU is often used to reflect codon bias. In the chloroplast genome of *Z. officinale*, most of the preferred synonymous codons (RSCU > 1) possess A- or U-ending codons, except for trnL-CAA, which UUG encodes. Codons ending with A and/or U accounted for 71.2%, resulting in the bias for A/T bases [33]. However, most codons with an RSCU > 1 of the chloroplast genome of *Litsea* contained either an A- or G-terminal. By contrast, RSCU values for codons that ended with a C-terminal, such as CGC (Arg), UGC (Cys), CAC (His), and AGC (Ser), were relatively low [32,34].

Repeated sequences are involved in stabilizing and rearranging sequences in the cp genome. Repeated sequences can be used to construct molecular markers for plant identification and molecular evolutionary genetic analysis [37,38,39]. In the *F. simplicissima* m3 plastid, the majority of SSRs were found in the intergenic spacer regions rather than in the coding regions. They primarily consisted of AT subunits, which were similar to those in the cp genomes of angiosperms has been reported in previous works [40,41]. Besides, mono- and tri- nucleotide SSRs were more prevalent than any other type of SSRs in the studied *F. simplicissima* m3 species. It was reported that large and complex repeats also involved the sequence rearrangement and the evolution of cp genomes [42,43]. The REPuter analysis resulted in 39 dispersed repeats distributed mainly in the intergenic spacer and intron sequences of the *F. simplicissima* m3 plastome. This number was much higher than that of the recently published *F. simplicissima* cp genome [13]; however, the repeat type content performed similarly to the prevalent palindromic and forward repeats.

The IR regions’ size of the *F. simplicissima* Lour m3 was 24.4 kb, which was consistent with the data observed in most angiosperm cp genomes (20–28 kb) [44]. In most land plants, the cp genome commonly displays some significant variations, such as gene loss, sequence inversion, and expansion/contraction of the IR regions, which lead to length differences among cp genomes [45,46,47]. The sizes of the *Ficus* cp genomes differed with some remarkable variations in the junction regions. According to the Irscope result, there was a significant IR contraction in the *F. simplicissima* m3 plastome, which was a decrease in size from about 25.8 kb to 24.4 kb. The boundaries between the SSC and the IRs were similar among the *Ficus* species. Besides, the junctions between LSC and IRs of *Ficus* plastomes were commonly located within the *rps19* gene (Figure 5). However, there were several cp genomes reported that the *rps19* gene does not extend into the IR region [48,49]. The studied cp genome witnessed an absence of this gene in the JLA region, which might be the cause of IR contraction. On the other hand, a difference of two bp in length between IRa (24,421 bp) and IRb (24,423 bp) was predicted. The cp genome annotation tools differed notably in the number of sequences identified, in which the IR regions differed in length [50]. The *ndhF* gene extended over the JSB and overlapped with the *ycf1* gene in half of the compared genomes, which had also been observed in some published cp genomes [13,51].

This study demonstrates that the variability of the *ycf1*, *rpoC2*, *rpoC1*, *ycf2*, and *ndhF* gene regions of *F. simplicissima* species is higher than that of the five compared species. Therefore, these gene regions can be used to elucidate the phylogenetic relationships within the *Ficus* genus. The *Ycf1*, *ycf2*, *rpoC2*, and *ndhF* gene regions have been confirmed to be the most disparate regions in the *Ficus* cp genome of the Moraceae family [52]. For the Asteraceae family, the two gene regions *rpoC1* and *ycf1* were also found to be the most distinct of the cp genomes of this family [53].

In the *Ficus* cp genomes, the sequence regions located on the genes *trnL-ccsA-ndhD*, *trnC-GCA*, and *rrn23S*, along with three intergenic spacer regions including *rps16-trnQ*, *rps14-psaB and clpP-psbB*, were highly variable regions and *trnL-ccsA-ndhD* sequence regions had the highest nucleotide diversity value (>0.015). Thus, these regions could be potential DNA barcodes for species identification.

Chloroplast genome data play an important role in species definitions due to the application of organelle-based “barcodes” to reveal the phylogenetic relationships among species [40]. The Moraceae family is known to have about 1100 species of 40 genera and they are distributed mainly in tropical and subtropical regions [54]. Currently, the study of Moraceae’s molecular evolutionary and phylogenetic analysis is limited, especially the species of the *Ficus* genus. Recently, Huang et al. (2022) evaluated the cp genomes of ten species in the *Ficus* genus. The results showed that *Morus* and *Ficus* had a close relationship compared to other genera of the Moraceae family with high bootstrap values [13,55]. In this study, the phylogenetic relationships by ML analyses were constructed based on two approaches, the complete cp genome sequence and the *psbA-trnH* intergenic spacer region. The two phylogenetic trees had congruent topologies (Figure 4). The outgroups, *Chaetachme*, *Broussonetia*, and *Morus*, clustered into monophyletic clades and were sisters to *Ficus*. Our study has made it clear that the complete cp genome of *F. simplicissima* can support genus-level identification in the Moraceae family.

At the species level, in Figure 4, *Ficus* were divided into two subgroups and the voucher in the same species clustered together to a certain degree. *F. simplicissima* and *F. hirta* diverged, indicating the genetic divergence between these two species and others, followed by *F. variolosa*. The sister relationships of the subgroups in the *Ficus* genus are consistent with previous reports [13]. According to Burgess et al. (2011), up to 97% accurate identification of Canadian temperate plant samples was possible based on five gene regions *rbcL*, *matK*, *rpoC1*, *psbA-trnH*, and *atpF-atpH* [56]. Research by Newmaster et al. (2008) showed that using *matK* and *psbA-trnH* data could identify more than 94% of species in the Myristicaceae family [57]. The study’s results using the *psbA-trnH* marker to identify the above plant objects are the basis for us to choose *psbA-trnH* for phylogenetic analysis of *Ficus* species. These findings supported the Berg classification system [58]. Thus, the *psbA-trnH* gene region sequence of the cp genome may be a potential candidate for chloroplast DNA barcoding for species-level identification in the *Ficus* genus. These recommendations contribute to species and genera identification based on cpDNA molecular markers and morphological support methods and help to further illustrate a monophyletic group within the Moraceae family.

## 5. Conclusions

In this study, the complete cp genomes of *F. simplicissima* were sequenced using the Pacbio sequel platform and compared with five other species of the *Ficus* genus in the Moraceae family. The cp genome of *F. simplicissima* m3 with a size of 160,321 bp contains 127 genes, including 85 protein-coding genes, eight rRNA genes, and 34 tRNA genes. Of these, 18 genes were duplicated in the IR region, and 21 contained introns. The molecular evolutionary genetics analysis results based on the complete cp genome and *psbA-trnH* intergenic region established the phylogenetic tree. The genetic relationship of *F. simplicissima* to other species in the Moraceae family was determined. The complete cp genome of *F. simplicissima* can support genus-level identification in the Moraceae family and the *psbA-trnH* gene region of the cp genome may be a potential candidate for chloroplast DNA barcoding for species-level identification in the *Ficus* genus.

## Figures and Tables

**Table 1:** Summary of the chloroplast genome of *Ficus simplicissima* m3 species.

Genome Size (bp)	160,321
LSC size (bp)	91,346
SSC size (bp)	20,131
IR size (bp)	24,423
GC content (%)	35.9
No. of genes	127
No. of PCGs	85
No. of tRNA	34
No. of rRNA	8

## Data Availability

Instances and data are available under request.

## Supplementary Materials

The following supporting information can be downloaded at: https://www.mdpi.com/article/10.3390/cimb45020067/s1, Figure S1: The long repeat sequences in the cp genome of *Ficus simplicissima* m3; Table S1: Gene contents in the complete chloroplast genome of *Ficus simplicissima* m3.
