# Supplementary material for: Analysis of the Chloroplast Genome of Ficus simplicissima Lour Collected in Vietnam and Proposed Barcodes for Identifying Ficus Plants
Source: Curr Issues Mol Biol. 2023 Jan 27;45(2):1024–36. doi: 10.3390/cimb45020067 (PMC9955830; doi:10.3390/cimb45020067)
Supplement: Supplementary file 1 [file cimb-45-00067-s001.zip › Supplementary.pdf]

SUPPLEMENTARY 1

Table S1. Gene contents in the complete chloroplast genome of *Ficus simplicissima* m3

Classification of Genes		Name of Genes	Number
RNA genes	Ribosomal RNAs	*rrn4.5(x2), rrn5(x2), rrn16(x2), rrn23(x2)*[a]	8
	Transfer RNAs	*trnA_UGC(x2)*[a], *trnC_GCA, trnD_GUC, trnE_UUC, trnF_GAA, trnfM_CAU, trnG_GCC, trnG_UCC, trnH_GUG, trnI_CAU(x2), trnI_GAU(x2)*[a], *trnL_CAA(x2), trnL_UAA, trnL_UAG, trnM_CAU, trnN_GUU(x2), trnP_UGG, trnQ_UUG, trnR_ACG(x2), trnR_UCU, trnS_GCU, trnS_GGA, trnS_UGA, trnT_UGU, trnV_GAC(x2), trnW_CCA, trnY_GUA*	34
Protein-coding genes	Photosystem I	*psaA, psaB, psaC, psaI, psaJ*	5
	Photosystem II	*psbA, psbB, psbC, psbD, psbE, psbF, psbH, psbI, psbJ, psbK, psbL, psbM, psbT, psbZ*	14
	Cytochrome	*petA, petB*[a], *petD*[a], *petG, petL, petN*	6
	ATP synthase	*atpA, atpB, atpE, atpF*[a], *atpH, atpI*	6
	Rubisco	*rbcL*	1
	NADH dehydrogenease - like complex	*ndhA*[a], *ndhB(x2)*[a], *ndhC, ndhD, ndhE, ndhF, ndhG, ndhH, ndhI, ndhJ, ndhK*	12
	Ribosomal proteins - small units	*rps11, rps12(x2)*[a], *rps14, rps15, rps16*[a], *rps18, rps19, rps2, rps3, rps4, rps7(x2), rps8*	15
	Ribosomal proteins - large units	*rpl14, rpl16*[a], *rpl2(x2)*[a], *rpl20, rpl22, rpl23(x2), rpl32, rpl33, rpl36*	11
	RNA polymerase	*rpoA, rpoB, rpoC1*[a], *rpoC2*	4

	Miscellaneous	*accD, ccsA, cemA, clpP1^b, matK*	5
	Hypothetical chloroplast reading frames (ycf)	*ycf1(x2), ycf2(x2), ycf3^b, ycf4*	6
Total			127

Notes: Genes marked with the sign are the gene with a single (a) or double (b) introns and duplicated genes (×2).

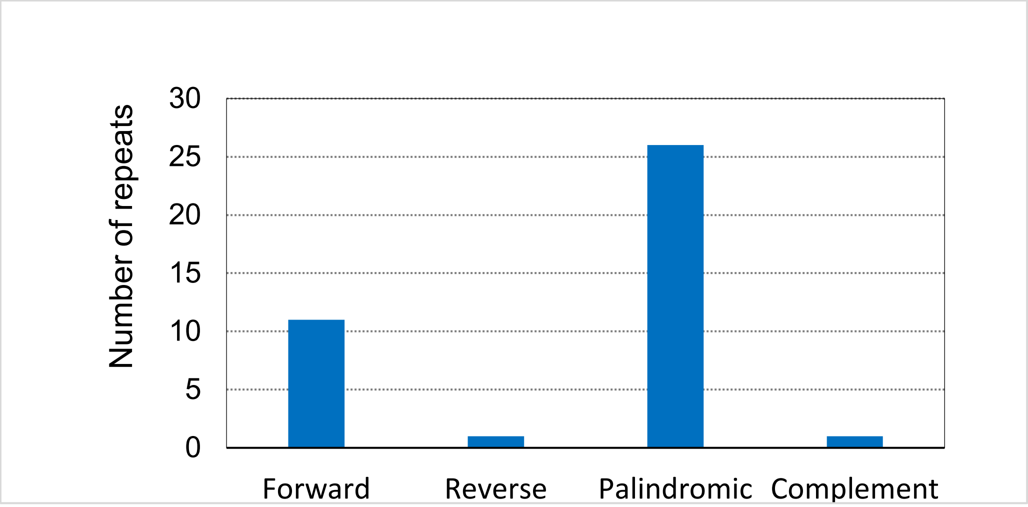

Figure S1. The long repeat sequences in the cp genome of *F. simplicissima* m3
